# Information about management of chronic drug therapies prescribed at hospital discharge: does it affect patients’ knowledge and self-confidence?

**DOI:** 10.1186/s12913-018-2895-2

**Published:** 2018-02-12

**Authors:** Claudia Pileggi, Emilia Caligiuri, Carmelo G. A. Nobile, Maria Pavia

**Affiliations:** 1 Department of Health Sciences, University of Catanzaro “Magna Græcia”, Via Tommaso Campanella, 88100 Catanzaro, Italy; 2 Department of Pharmacy, Health and Nutritional Sciences, University of Calabria, Arcavacata di Rende, 87036 Cosenza, Italy

**Keywords:** Chronic drug therapies, Discharge counseling, Patient safety, Continuity of care

## Abstract

**Background:**

Hospital stay represents the opportunity for a change of therapy, about which patients may not know indications, contraindications, and mode of administration, which may lead to dosing errors, drug interactions, side effects, etc. It is therefore vital to communicate appropriate information to the discharged patient with a new prescription drug. The purpose of the study was to evaluate: 1) how communication about new chronic therapies is managed at hospital discharge and what kind of information is provided to patients; 2) to what extent patients are aware and confident in the management of these medications; 3) whether the way communication is provided affects patients’ awareness and self-confidence in the management of these therapies.

**Methods:**

Participants were adult patients who were prescribed at least one new chronic medication at hospital discharge. A telephone interview after hospital discharge was performed to assess whether or not hospital healthcare personnel had given information about prescribed therapies and which aspects of therapies had been object of information.

**Results:**

Five hundred thirty patients were interviewed. 67.7% reported having received counseling by the hospital physician, while 32.3% by discharge form. Basic information on treatment was provided to the great majority of patients, whereas only few patients reported to have been informed about eventual side effects and related behavior in case of side effects.

**Conclusions:**

Several aspects of patients’ knowledge and self-confidence on long term medications prescribed at hospital discharge need to be improved and the way communication is provided has a crucial role in the empowerment of patients in the management of these medications.

## Background

Chronic conditions are health problems that require ongoing management over a period of years or decades, and cover a wide range of conditions that go beyond the conventional definition of chronic illness, such as cardiovascular and respiratory diseases or diabetes. They also extend to some persistent communicable diseases (e.g. HIV/AIDS), to certain mental disorders (e.g. depression), and to cancer [1].

Advances in healthcare have led to growing numbers of people surviving with chronic illness. At the same time, the significant growth of elderly population led to the emergence of new needs associated with increasing number of those with chronic health problems because of accumulated exposure to chronic disease risk factors over their lifetime. The common theme of chronic illness is that these conditions require complex models of care, over an extended time period that involves coordinated inputs from a wide range of health professionals and access to essential medicines and monitoring systems, all of which need to be optimally embedded within a system that promotes patient empowerment.

Several theoretical frameworks and delivery models were designed to approach patients with chronic diseases and various levels of healthcare need [2–6]. Perhaps the most influential framework has been the Chronic Care Model (CCM) developed by Wagner and colleagues [7] and adapted by WHO [8] in a global perspective. It was based on the premise that high-quality chronic care is characterized by productive interactions between the practice team and patients, involving assessment, self-management support, optimization of therapy and follow-up.

A key role in the appropriate management of chronic diseases care is played directly by the patient him/herself, through effective adherence to a complex therapeutic pathway that mostly relies on chronic assumption of drugs, lifestyle changes, and follow-up controls. Similarly, patients play a substantial role in the safety of this process of care, by helping to reach an accurate diagnosis, by ensuring that treatment is appropriately administered, monitored and adhered to, by identifying side effects or adverse events and taking appropriate action [9]. The safety and effectiveness of hospital clinical pathways has long been studied and process and outcome indicators have been developed to monitor hospital performance [10–13], whereas less attention has been devoted to outpatient processes of care, particularly when therapies are mostly in charge of patients themselves.

In patients with chronic diseases, hospital stay frequently represents the opportunity for a prescription of new drugs, about which patients may not know indications, contraindications, and mode of administration, which may lead to dosing errors, drug interactions, side effects, but also failure to achieve the expected therapeutic targets. Moreover, it has been hypothesized that primary non-adherence, defined as failure to fulfill a newly prescribed medication, could be related to poor communication between the inpatient medical team and the patient, as well as with the primary care physician (PCP) [14].

At the time of discharge, the patient may obtain information and data about new prescribed medication from various sources such as counseling or written information [15, 16], but doctors, nurses and pharmacists are the main source of this information [8]. It is therefore vital to communicate appropriate information to the patient discharged with a new prescription drug [9]. Proper notification to the person increases the degree of adherence to treatment [17, 18], which, despite being influenced by psychological, social, cultural and environmental factors, is seriously undermined if the patient has not been provided the necessary information or if it has been perceived in a distorted manner [19].

Therefore, the purposes of the study were to evaluate: 1) how communication about new chronic therapies is managed at hospital discharge and what kind of information is provided to patients; 2) to what extent patients are aware and confident in the management of these medications; 3) whether the way communication is provided affects patients’ awareness and self confidence in the management of these therapies.

## Methods

The study was designed as cross-sectional. The eligible population was selected among patients admitted for ordinary hospitalization in one academic hospital (n. 250 beds) and one non-academic hospital (n. 450 beds) located in Catanzaro, Italy. The selection process of eligible patients consisted of two phases. In the first phase, that was carried out during hospital stay, potentially eligible patients had to fulfill the following inclusion criteria: 1) age > 18; 2) admission in a medical ward (Cardiology, Internal Medicine, Nephrology, Hepatology, Respiratory Diseases, Geriatrics, Metabolic Diseases) at least 48 h before selection. All of these patients were invited to participate in the study during hospitalization by physicians not involved in clinical care, that explained them the purpose and the design of the study, and a written informed consent to participation was asked to those who were willing to take part in the study. In the second phase of the selection process the medical records of patients that had provided informed consent were reviewed after patients’ discharge in order to select those who were prescribed, at time of discharge, at least one new chronic medication, that was not present in their treatment upon admission. A medication was considered chronic if prescribed for 30 days or longer. Patients who were prescribed dose modifications of existing drugs in the treatment or substitution by another equivalent drug and those that were potentially suffering of a cognitive impairment, as shown by anamnestic data or psychological/psychiatric consultations reported in the medical record, were excluded from the study.

Consultation of medical records enabled collection of further information on hospitalization (ward and diagnosis at admission and at discharge).

Patients included in the study after the two-phases selection process underwent a structured telephone interview within 3 weeks after hospital discharge.

In the telephone interview, if more than one new medication was prescribed, information was collected on the drug that had the longer length of prescription. It was first asked whether or not hospital healthcare personnel had given information about prescribed therapies at discharge, and separated questions were asked according to source of information. In patients who reported to have been informed by hospital healthcare personnel, an in depth interview was performed to investigate which aspects of therapies had been object of information to patients: reasons for the prescription of the new drug, mode (dosage and route of administration) and time of drug intake, duration of treatment, necessity and frequency of periodic controls, behaviour in case of forgotten medication intake or in case of having taken the wrong dose of medication, necessity to avoid concomitant use of the drug with other drugs and/or food, side effects and behavior in case of occurrence of side effects, possibility of discontinuing therapy as a consequence of medical condition improvement and consequences of not taking the therapy. In those patients who reported to have not been informed by hospital healthcare personnel about prescribed medications eventual other sources of information were retrieved (PCP, private specialist, pharmacist, discharge form, internet, other patients taking the same medication, etc.).

Moreover, in all participant patients, regardless of the source of information, medication knowledge and patient perceived self-confidence were assessed by the questions drawn from the Okere-Renier Survey instrument [20]. Finally, the need for more information and the favourite person to contact in case of doubt about therapy, were also investigated.

A sample size of about 500 patients was calculated to have a 80% power and an α of 0.05 to detect a 15% difference in knowledge about new medications between patients who received counseling by healthcare personnel as compared to discharge form.

The study was conducted from January 2012 to February 2013.

### Statistical analysis

Data were analyzed using the Stata software program [21]. Continuous variables were compared using the two-tailed Student’s t test. Categorical variables were compared using the Pearson’s chi-square test or two-tailed Fisher’s exact test, when appropriate. One model was developed using multiple logistic regression analysis to identify the variables related to the way information was provided at discharge (0 = letter of hospital discharge, 1 = hospital specialist). The explanatory variables included in model were the following: sex (0 = male, 1 = female), hospital (0 = academic, 1 = non academic), age groups (0 = < 65, 1 = ≥65), marital status (0 = other, 1 = married), working activity (0 = unemployed or retired, 1 = employed), discharge ward (0 = general medicine, 1 = specialist care), number of new prescription drugs (0 = one prescription, 1= > one prescription), need for more information about therapy (0 = no, 1 = yes), knowledge about behaviour in case of having taken the wrong dose of medication (0 = no, 1 = yes), knowledge about duration of treatment with new medication (0 = no, 1 = yes), knowledge about possible side effects (0 = no, 1 = yes), knowledge about behaviour in case of having forgotten medication intake (0 = no, 1 = yes), favourite person to contact in case of doubt about therapy (0 = PCP, 1 = hospital or private specialist). Adjusted odds ratio (OR) and 95% confidence intervals (CI) were calculated.

The study protocol was approved by the Ethics Committee of the “Mater Domini” Hospital of Catanzaro (Italy) (15/12/2011).

## Results

During the study period, 558 patients were discharged with a prescription of a new drug. Of these, 12 patients (2.1%) refused to be interviewed, 4 (0.7%) were excluded because they were re-hospitalized, 1 (0.2%) because of serious health conditions, and 11 (2%) were not available after 10 calls. Therefore the results are reported for 530 patients, for a 95% response rate, that were interviewed within 3 weeks after discharge. The average age of the participants was 63.3 years (±13.7 years), men constituted 57% of the sample. The majority of patients were married (77.4%) and 24.2% had acquired more than 8 years of education. 76.8% of the patients were unemployed or retired.

Hospital admission was related to cardiovascular diseases in 47.5% of patients, and to liver and kidney diseases in 15.3% and 11.2% of patients, respectively. Other diagnoses were involved in 26% of the admissions.

67.7% reported having received counseling about their medications by the hospital physician at the moment of discharge, while for the remaining 32.3% the only source of information was the discharge form. Among these, after discharge, 6 patients sought further clarification about new prescribed drugs to PCPs, 2 patients to private specialist physicians and 2 to pharmacists. Table 1 reports the information about new prescribed drug(s) provided to patients by hospital physicians at discharge. Basic information on treatment, such as reason, dosage and route of administration, time and duration of treatment, necessity and frequency of controls, was provided to the great majority of patients, just over half received information about the consequences of not taking the therapy and about avoiding concomitant use of the drug with other drugs and/or foods, whereas only few patients reported to have been informed about eventual side effects and related behavior in case of side effects, as well as in case of having forgotten or having taken the wrong dose of medication. Table 2 reports general characteristics of patients, actual knowledge about new prescriptions received and disaggregated knowledge according to source of information (hospital physician at discharge or discharge form). Virtually all patients are aware of mode, time and duration of administration of the new prescribed drug, whereas lack of knowledge is related to behaviors to have when one forgets or accidentally takes wrong doses of the new drug; moreover, among those who reported to know how to behave, the most frequently reported response was to ask to the physician, particularly the PCP (54.9%). Even a lower percentage of patients (8.5%) is aware of possible side effects, whereas 41.5% reported to need more information about the new prescription, particularly about duration (33%), side effects (26.8%), and mode of administration (21.4%).

**Table 1:** Information about new prescribed drug provided to patients by hospital physician at discharge

Characteristic	Number	Percent
Reasons for the prescription of new drugs	320	89.1
Mode of drug intake (dosage and route of administration)	359	100
Timing of drug intake	358	99.7
Duration of treatment	291	81
Necessity of periodic controls	324	90.2
Frequency of periodic controls	317	88.3
Behavior in case of forgotten drug intake	34	9.5
Behavior in case of having taken the wrong dose of medication	15	4.2
Avoid concomitant use of the drug with other drugs and / or foods	205	57.1
Side effects	35	9.7
Behavior in case of occurrence of side effects	29	8.1
Possibility of discontinuing therapy as a consequence of improvement	46	12.8
Consequences of not taking the therapy	207	57.7

**Table 2:** Distribution of source of information about the new prescription drug according to characteristics and knowledge of patients

Characteristic			Hospital physician		Discharge form	
	N(530)	%	N	%	N	%
Sex						
Male	302	57	188	62.2	114	37.8
Female	228	43	171	75	57	25
			χ^2^ = 9.66, 1 df*, p* = 0.002			
Age						
Mean ± SD	63.3 ± 13.7		62.9 ± 13.9		64.1 ± 13.3	
			*t* = 0.87, 528 df, *p* = 0.387			
Marital status						
Married	410	77.4	278	67.8	132	32.2
Other	120	22.6	81	67.5	39	32.5
			χ^2^ = 0.004, 1 df*, p* = 0.950			
Education level, years of schooling						
None	53	10	32	60.4	21	39.6
5	166	31.3	115	69.3	51	30.7
8	183	34.5	120	65.6	63	34.4
≥ 13	128	24.2	92	71.9	36	28.1
			χ^2^ = 2.88, 3 df*, p* = 0.410			
Working activity						
Unemployed/ Retired	407	76.8	273	67.1	134	32.9
Artisan/lower managerial	69	13	46	66.7	23	33.3
High professional and managerial	54	10.2	40	74.1	14	25.9
			χ^2^ = 1.11, 2 df*, p* = 0.574			
Living condition						
Alone	40	7.5	28	70	12	30
Other	490	92.5	331	67.5	159	32.5
			χ^2^ = 0.1, 1 df*, p* = 0.750			
Academic hospital						
Yes	296	55.8	242	81.8	54	18.2
No	234	44.2	117	50	117	50
			χ^2^ = 60.31, 1 df*,* = < 0.001			
Discharge ward						
General medicine	161	30.4	111	68.9	50	31.1
Specialistic care	369	69.6	248	67.2	121	32.8
			χ^2^ = 0.15, 1 df*, p* = 0.694			
Number of new prescription drugs						
1	197	37.2	145	73.6	52	26.4
> 1	333	62.8	214	64.3	119	35.7
			χ^2^ = 4.94, 1 df*, p* = 0.026			
Reported behavior about mode of administration of the new drug						
Correct	527	100.0				
Not correct	0	–				
Reported behaviour about time of administration of new drug						
Correct	479	99.8				
Not correct	1	0.2				
Knowledge about duration of treatment						
Correct	348	96.7	254	73	94	27
Not correct	12	3.3	4	33.3	8	66.7
			χ^2^ = 8.98, 1 df*, p* = 0.003			
Knowledge about behavior in case of having forgotten medication intake						
Yes	163	30.8	123	75.5	40	24.5
No	367	69.2	236	64.3	131	35.7
			χ^2^ = 6.43, 1 df*, p* = 0.011			
Knowledge about behaviour in case of having taken the wrong dose of medication						
Yes	127	24	100	78.7	27	21.3
No	403	76	259	64.3	144	35.7
			χ^2^ = 9.25, 1 df*, p* = 0.002			
Knowledge about possible side effects						
Yes	45	8.5	33	73.3	12	26.7
No	485	91.5	326	67.2	159	32.8
			χ^2^ = 0.71, 1 df*, p* = 0.401			
Favourite person to contact in case of doubt about therapy						
PCP	291	54.9	173	59.4	118	40.6
Hospital specialist	205	38.7	167	81.5	38	18.5
Private specialist	34	6.4	19	55.9	15	44.1
			χ^2^ = 29, 2 df*, p* = < 0.001			
Need for more information about the new medication						
Yes	220	41.5	117	53.2	103	46.8
No	310	58.5	242	78.1	68	21.9
			χ^2^ = 36.45, 1 df*, p* < 0.001			

The numbers that do not add to 530 are due to missing data for the variable

At univariate analysis, patients who correctly reported the duration of therapy, who were confident about the behavior to have in case they forget or take a wrong dose of the prescribed drug were significantly more likely to have been informed by the hospital physician at discharge, as well as females, those who were discharged by an academic hospital and those who do not feel they need more information about the new prescribed drug. As expected, the favourite person to contact in case of doubt about therapy was significantly more likely to be the hospital or private specialist (Table 2). These results were substantially confirmed by multivariate analysis, except for the knowledge about behavior in case of having forgotten medication intake (Table 3).

**Table 3:** Logistic regression model results

Outcome: Having received information about the new treatment by the hospital physician				
Variable	OR	SE	95% CI	p
Log likelihood = − 275.2, Chi square = 116.2, *p* < 0.0001				
Hospital				
Academic (reference)	1.00			
Non Academic	0.28	0.06	0.18–0.43	< 0.001
Need for more information about therapy				
No (reference)	1.00			
Yes	0.42	0.93	0.28–0.65	< 0.001
Knowledge about behaviour in case of having taken the wrong dose of new medication				
No (reference)	1.00			
Yes	2.02	0.54	1.19–3.42	0.009
Gender				
Male (reference)	1.00			
Female	1.83	0.43	1.16–2.89	0.010
Favourite person to contact in case of doubt about therapy				
PCP (reference)	1.00			
Hospital or private specialist	1.67	0.37	1.08–2.59	0.021
Knowledge about duration of treatment with new medication				
No (reference)	1.00			
Yes	1.62	0.4	1.00–2.61	0.049
Age group, years				
< 65 (reference)	1.00			
≥ 65	1.43	0.36	0.88–2.34	0.148
Marital status				
Other (single, separated, etc.) (reference)	1.00			
Married	1.35	0.35	0.81–2.26	0.245
Number of new prescription drugs				
1 (reference)	1.00			
> 1	0.79	0.18	0.5–1.22	0.288
Working activity				
Unemployed or retired (reference)	1.00			
Employed	1.37	0.42	0.75–2.5	0.301
Knowledge about possible side effects	*Backward elimination*			
Discharge ward	*Backward elimination*			
Knowledge about behavior in case of having forgotten medication intake	*Backward elimination*			

## Discussion

Although transitions across health care settings are recognized to be vulnerable periods for the correct adherence to newly prescribed therapies, only few studies have addressed in depth how to overcome post hospital discharge medication problems, particularly for patients who are prescribed new drugs in the hospital and have to manage chronic therapies at home.

Our study has tried to explore one of the crucial factors involved in the correct continuity of care after hospital discharge, that is communication on new drug chronic therapies prescribed at hospital discharge.

The first research question investigated how communication about new chronic therapies is managed at hospital discharge and what kind of information is provided to patients and the results revealed that there are only two ways information is provided: direct counselling by the hospital physician in about two thirds of patients and from the discharge form in the remaining cases. It should be noted that in Italy, although nurses are an important source of information, generally physicians are reluctant to rely on other healthcare professionals (nurses, pharmacists) to convey medication related information. This may be because physicians consider themselves to be the primary source of patient information and, on the other hand, patients prefer to be informed by physicians, as in other countries [22, 23].

About the finding that one third of discharged patients did not receive any counselling about new prescribed long term drugs, it should be noted that the discharge form is a compulsory part of the discharge process in Italy including information about the admission, diagnosis at discharge, comorbidities and the list of prescribed medications. The dose and the mode of intake of each medicine are noted along with the changes in medication after admission, whereas no information is reported about medicines side effects or interaction. The discharge form is given to the patient at discharge and a copy is addressed to the PCP that will take care of the discharged patient. This result is a concern, as already highlighted by Toren et al., that found that only 40% of patients discharged from hospital had received counselling and that it was associated with knowledge on medications [24]; moreover Coleman et al. reported that discrepancies between prescribed medication and actual old patients’ behaviour were also related to system-associated factors, such as conflicting information from different informational sources [25]. Although alarming, this finding was not surprising; in a study conducted by some of us to evaluate the adaptability of the Joint Commission on Accreditation of Healthcare Organizations (JCAHO) and the Centers for Medicare & Medicaid Services (CMS) quality indicators in our geographical area we found that rates regarding adherence to discharge instructions in heart failure patients, that is one of the patient safety indicators, was “noticeably intangible” [26], thus confirming low attention to counseling at discharge in our hospitals. The investigation of quality and extent of information provided to patients showed that counselling at discharge performed by hospital physicians on new prescribed therapies dealt mainly on the rationale, dosing, route and timing of medications administration, whereas potential problems and/or actions to take in presence of errors or side effects of medications were not discussed at discharge. This finding has already been reported, and the Authors highlighted that health care professionals are reluctant to discuss eventual problems related to medications, since they fear that this kind of information may represent a barrier to adherence [15]. However, patients may not be satisfied by the absence of this information, since they want to be informed about risks of medications [17, 22, 27, 28], and it has been reported that if patients are not satisfied about information received on prescribed therapies, there may be a negative impact on adherence to medications [29, 30, 31].

The second research question explored to what extent patients were aware and confident in the management of new prescribed medications and the findings of the study demonstrated that there is a very close agreement between the information provided and the self-confidence of patients, as well as a gap of knowledge in the areas that were not the object of counselling, such as drugs side effects or the behaviours to have if one forgets or takes wrong doses of the new drugs. This finding has been already reported by Thoren et al., who highlighted the risk that inadequate knowledge on these issues may limit the empowerment of patients representing a barrier to the autonomous management of therapies after discharge [24]. This is confirmed by our findings showing that even patients that feel confident on how to behave reported that they would refer to their PCP.

The main finding of our study, exploring the hypothesis that the way communication is provided may affect patients’ awareness and self-confidence in the management of new chronic drug therapies, showed that counselling by the hospital physician was significantly associated with higher knowledge and self-confidence. Although, as already discussed, the quality of counselling might be improved, our study demonstrated the effectiveness of the source of information for several aspect of knowledge, such as duration of therapy and behaviour to have in case of forgotten or wrong dose of drug taken. A certain degree of empowerment of patients was expressed by the finding that those who received information by the hospital physician significantly more frequently declared they did not need more information about the new prescribed drug, that was reported to be also the favourite person to contact in case of doubts about the therapy. Similar results have been reported by Micheli et al., who found that patients who had received information on long term therapies during hospitalization were significantly more likely to have knowledge about reasons for taking them [32], and Alkatheri et al., who reported that education level of patients and previous counselling were positively linked to medication knowledge [33]. Counselling was also associated to academic setting, but, since we evaluated only two hospitals, a cautious approach to the generalization of this finding is warranted, although it may be hypothesized that younger physicians, that attend their post graduate residency in the academic hospitals, may be more motivated since counselling to patients is among the learning goals of their training process. The only patient-related characteristic that showed to be associated to information provided by physician was female sex. We did not explore whether counselling was spontaneously administered by physicians or it was at request of the patient; however, since from previous research we have indirectly found that women are more concerned about their health, since they rated their health less satisfactory compared to men [34], and were more likely to use internet for health related issues [35], we may only hypothesize that they were more likely to stimulate hospital physicians to provide more detailed information about new prescribed drugs.

In a study assessing satisfaction on information about medicines provided to cardiac patients during hospitalization, the Authors could not discern whether written or in-person information was more effective [15], whereas in our study the peculiar Italian discharge process that includes a discharge form addressed to PCPs allowed us to assess that when written information is supplemented with physician-patient counseling the resulting patients’ medication knowledge is significantly increased.

It is well-known that the hospital discharge process represent a crucial step for the potential consequences in the management of chronic patients in other healthcare settings. Our results clearly indicate that the discharge form as the only tool to inform patients about new medications is absolutely inadequate, and that counselling by physicians significantly improves patients’ knowledge and self-confidence. It seems therefore useful to promote in our context a more extensive involvement of other hospital professional figures, such as nurses and pharmacists in the delicate discharge process of chronic patients.

### Limitations

Some potential limitations of the present study need to be acknowledged. We collected data in two hospitals and concern about generalizability of our results may arise. However, although we cannot exclude that our results pertain only to our area, we may be quite confident that the context we have studied may be generalized at least to the Southern Italy hospitals. Moreover, data were based on medical record documentation and on patients self-reporting; however, we do not think that method of data collection may represent a problem in this case because self-reporting is the only way to collect information about knowledge and self-confidence in the management of drug prescriptions. Third, as is the case of all surveys, another limitation is the potential recall bias; nonetheless this was a minor issue considering the restricted 3 weeks period between discharge and telephone interview. It should also be noted, however, that the 95% response rate was extremely satisfactory and reduces one major potential source of bias in the results, that is sample representativeness. Finally, we used self-reported knowledge on several aspects of new prescribed drugs as an indicator of effective communication at discharge and as a prerequisite for effective adherence to prescribed therapies, whereas we did not directly explore communication strategies performed by physicians, nor actual adherence to treatments.

## Conclusions

In conclusion, our results have demonstrated that several aspects of patients’ knowledge and self-confidence on long term medications prescribed at hospital discharge need to be improved and that the way communication is provided has a crucial role in the empowerment of patients in the management of these medications. Since adequate knowledge has been related to adherence to medications, efforts are needed to select and introduce in the hospital settings effective communication and counseling strategies.

## Abbreviations

CI: Confidence interval
CMS: Center for Medicare & Medicaid Services
JCAHO: Joint Commission on Accreditation of Healthcare Organizations
OR: Odds ratio
PCP: Primary care physician
